# Beyond vision: economic toll of untreated mental health disorders in ophthalmic patients

**DOI:** 10.1192/bjo.2025.10870

**Published:** 2025-10-17

**Authors:** Cansu Yüksel Elgin, Ceyhun Elgin

**Affiliations:** Department of Ophthalmology, Istanbul University-Cerrahpasa, Istanbul, Turkey; Department of Economics, Bogazici University, Istanbul, Turkey

**Keywords:** Mental health economics, vision impairment, healthcare access disparities, ophthalmic patients, health policy interventions

## Abstract

**Background:**

Mental health disorders such as depression and anxiety are highly prevalent among ophthalmic patients, particularly those with progressive vision impairment. Despite the strong interconnection between mental health and vision-related disabilities, mental health support remains underintegrated into ophthalmic care. The economic burden of untreated mental health conditions in visually impaired patients is understudied, particularly in middle-income countries such as Turkey and Bulgaria.

**Aims:**

This study aims to examine the economic impact of untreated mental health disorders among ophthalmic patients, focusing on financial burden, healthcare access disparities and quality of life outcomes. In addition, the study compares barriers to mental healthcare across ophthalmic conditions and between Turkey and Bulgaria.

**Method:**

A qualitative study was conducted using structured surveys and in-depth interviews with 214 ophthalmic patients (107 in Turkey, 107 in Bulgaria). Mental health symptoms were assessed using the Patient Health Questionnaire-9 (for depression) and Generalized Anxiety Disorder-7 (for anxiety) scales. Thematic analysis was applied to qualitative responses.

**Results:**

Over 50% of participants exhibited moderate-to-severe depression and anxiety, with diabetic retinopathy and retinal disease patients experiencing the highest distress levels. Financial barriers were more pronounced in Bulgaria, whereas long psychiatric wait times disproportionately affected retinal patients. Mental health stigma was higher in Bulgaria, limiting care access.

**Conclusions:**

Findings underscore the urgent need for integrating mental health services into ophthalmic care. Policy interventions should focus on financial support, stigma reduction and improved interdisciplinary care models to enhance mental health outcomes for visually impaired individuals.

Mental health disorders, including depression and anxiety, have increasingly been recognised as major public health challenges with significant economic implications. According to the World Health Organization (WHO), depression is now the leading cause of disability worldwide, affecting more than 280 million people, while anxiety disorders impact nearly 300 million individuals globally.^1^ The economic burden of these conditions is immense, encompassing direct medical costs, productivity losses and increased demand on social services.^2^ While much of the research on the economic impact of mental health conditions has centred on psychiatric and primary care settings, there is growing evidence that comorbid mental health disorders can exacerbate the burden of physical illnesses, including ophthalmic conditions.^3^

The intersection of mental health and ophthalmology is an area of growing concern. Vision impairment has been strongly associated with an increased prevalence of depression, anxiety and cognitive decline.^4^ Patients with age-related macular degeneration (AMD), glaucoma and diabetic retinopathy, for example, have significantly higher rates of depression than the general population.^5^ The psychosocial impact of vision loss includes reduced quality of life, social isolation and loss of independence, all of which contribute to poor mental health outcomes.^6^ Despite this well-documented relationship, ophthalmic care often neglects mental health screening, leading to an underestimation of the economic and social burden of these comorbid conditions.

The economic cost of untreated mental health disorders in ophthalmic patients is multi-dimensional and complex. Studies suggest that individuals with visual impairment and concurrent depression incur higher healthcare costs, both because of increased medical visits and greater reliance on supportive services.^7^ In addition, vision impairment combined with poor mental health is associated with reduced workforce participation and lower productivity levels, leading to economic losses at both the individual and societal levels.^8^ From a health policy perspective, failing to integrate mental health screening and intervention into ophthalmic care may result in greater long-term financial strain on public health systems.^9^

Despite the clear connections between vision impairment and mental health, there remains a lack of research on the specific economic consequences of untreated mental health disorders in ophthalmic patients. Most studies have focused either on the clinical impact of comorbid conditions or on the broader economic costs of mental health issues, and few have examined the economic burden at the intersection of ophthalmology and psychiatry.^10^ Moreover, research is often skewed towards high-income countries, with limited data from middle-income settings, such as Turkey and Bulgaria, which will be the main focus of the present study. Given that economic and healthcare structures vary significantly across regions, a comparative study is crucial to understanding the financial impact of mental health comorbidities in ophthalmic patients across different healthcare systems.

This study seeks to bridge the existing gaps in research by conducting a qualitative analysis of ophthalmic patients in Bulgaria and Turkey, with a particular focus on the economic burden of untreated mental health conditions. Utilising a combination of structured surveys and in-depth interviews, the research will provide a comprehensive understanding of the financial and healthcare challenges faced by visually impaired individuals who also struggle with mental health issues.

A key objective of this study is to investigate the prevalence of mental health symptoms among ophthalmic patients. Given the established connection between vision impairment and psychological distress, this research aims to quantify the extent to which conditions such as depression and anxiety affect individuals dealing with ophthalmic diseases. By capturing these data, the study will contribute valuable insights into the psychological dimensions of visual impairment and its broader implications.

Another crucial aspect of this research is examining how untreated mental health disorders influence healthcare costs and utilisation. Mental health conditions often exacerbate physical illnesses, leading to increased medical visits, higher treatment expenses and greater reliance on healthcare services. Through qualitative analysis, this study will explore how financial burdens shape healthcare decisions among ophthalmic patients and the extent to which untreated mental health concerns impact overall medical expenditures.

Furthermore, this study will analyse socioeconomic disparities in access to mental healthcare among individuals with vision impairments. Economic status, insurance coverage and geographic location are factors that may significantly influence the ability of patients to seek appropriate mental health support. By comparing responses from Turkey and Bulgaria, the research aims to highlight inequalities in access to mental healthcare and identify potential systemic barriers that prevent visually impaired individuals from obtaining the psychological care they need.

Finally, the study will explore policy implications for integrating mental health screening into ophthalmic practice. Given the substantial overlap between visual impairment and mental health disorders, it is essential to develop healthcare policies that incorporate routine mental health assessments within ophthalmology settings. This research will provide recommendations for policymakers and healthcare providers, advocating for interdisciplinary approaches that ensure comprehensive care for ophthalmic patients, ultimately reducing economic strain and improving patient well-being.

## Method

This study employs a qualitative research design using structured surveys and in-depth interviews to explore the economic toll of untreated mental health conditions in ophthalmic patients. The study was designed to gather in-depth perspectives on the financial and emotional burdens faced by patients with visual impairments and coexisting mental health issues.

The study was conducted in two countries, Turkey and Bulgaria, each representing distinct healthcare systems and socioeconomic environments. In Turkey, the surveys were conducted at the Hamidiye Etfal Training and Research Hospital, a medical institution with a high volume of ophthalmic patients in Istanbul. In Bulgaria, the surveys took place at the Medical University of Sofia’s clinics, ensuring access to a diverse patient population within an academic healthcare setting.

Surveys were administered in person by trained assistants with backgrounds in psychology and healthcare economics. Participants were informed about the voluntary nature of the study and provided written informed consent before participation. The inclusion criteria required that participants be at least 18 years old, diagnosed with a moderate-to-severe ophthalmic condition (e.g. glaucoma, diabetic retinopathy, macular degeneration or corneal disorder) and willing to discuss their mental health status. Individuals with acute psychiatric conditions requiring immediate intervention were excluded to ensure ethical considerations and participant safety.

At each site we used consecutive sampling during routine out-patient hours. In Turkey, every third eligible patient who registered at the ophthalmology desk on clinic days was approached by a trained psychology-economics research assistant; refusals were logged (9.8%) to track non-response. In Bulgaria, a similar procedure was followed in two alternating ophthalmic clinics to ensure weekday variability; the refusal rate was 11.2%. Although consecutive sampling enhances real-world representativeness, it may under-sample patients who attend outside standard hours or those requiring emergency ophthalmic care. We attempted to mitigate this bias by including two late-afternoon data-collection sessions per week at both sites. Finally, because mental health disclosure can be sensitive, some degree of underreporting of symptoms is possible despite the use of validated self-report scales and assurances of confidentiality.

The survey instrument consisted of a combination of standardised and open-ended questions. The Patient Health Questionnaire-9 (PHQ-9) and the Generalized Anxiety Disorder-7 (GAD-7) scales were incorporated to assess depressive and anxiety symptoms among ophthalmic patients. In addition, questions were designed to capture socioeconomic background, healthcare utilisation and perceived financial burden caused by mental health conditions. Open-ended questions allowed participants to describe their experiences accessing mental healthcare and their perspectives on potential policy interventions.

Data collection spanned over 2 months, with research assistants conducting structured interviews alongside survey administration. Patients were asked about their employment status, out-of-pocket medical expenses and any perceived barriers to accessing mental health services. Special attention was given to the role of healthcare insurance and government support in mitigating the economic impact of untreated mental health disorders.

Given the qualitative nature of the study, data analysis focused on thematic coding and narrative synthesis. Thematic analysis was used to identify key patterns in patient responses regarding financial burden, healthcare access and the psychological toll of vision impairment. Responses were categorised into overarching themes such as economic stress, lack of mental health integration in ophthalmic care and disparities in access to treatment.

Descriptive summary statistics were employed to present demographic data and mental health prevalence among respondents, but no inferential statistical analyses were conducted. The qualitative insights were then synthesised to inform policy recommendations and potential healthcare interventions.

The findings from this methodological approach aim to provide a comprehensive understanding of the challenges faced by ophthalmic patients in accessing mental healthcare and the resulting economic implications, offering evidence-based recommendations for policy reform.

A total of 214 participants were recruited for this study, with 107 participants from Turkey and 107 from Bulgaria. The age distribution ranged from 18 to 85 years, with a mean age of 58.3 years (s.d. = 14.7). Gender distribution was relatively balanced, with 112 female (52.3%) and 102 male (47.7%) participants.

Among the Turkish participants, 61.7% were aged 50 and above, while the Bulgarian sample had a slightly higher proportion of younger participants, with 48.2% under 50 years old. The most commonly diagnosed ophthalmic conditions included glaucoma (28.3%), diabetic retinopathy (27.1%), AMD (24.6%) and corneal disorders (20.0%). In addition, 82.2% of participants reported experiencing some level of financial difficulty in managing their healthcare costs.

Employment status varied, with 40.7% of respondents being retired, 29.3% employed and 30.0% unemployed. Health insurance coverage differed significantly between the two countries, with 91.5% of Turkish participants having some form of health insurance, compared with 78.2% in Bulgaria.

In addition, we conducted inferential statistical analyses to examine group differences in the prevalence of depression and anxiety, as well as financial and structural barriers to mental healthcare. Specifically, chi-squared tests were employed to determine whether mental health symptom severity (PHQ-9 and GAD-7 scores) varied significantly across different ophthalmic conditions. Similar tests were applied to assess differences in access-related barriers between Turkey and Bulgaria and across diagnostic subgroups. A two-tailed significance threshold of *p* < 0.05 was used for all statistical tests. These analyses were conducted using Stata version 18 for macOS.

## Results

This section presents the findings of the study in a detailed and structured manner. The data analysis focused on understanding the financial burden, mental health conditions and barriers to accessing mental healthcare among ophthalmic patients in these two countries. The qualitative data were analysed using thematic analysis following Braun and Clarke’s framework,^11^ identifying key themes emerging from participant responses. The findings are structured under the following sections: financial burden of untreated mental health conditions, prevalence and severity of mental health symptoms, impact on quality of life, barriers to seeking mental healthcare and comparative analysis between Turkey and Bulgaria.

The financial burden of untreated mental health conditions among ophthalmic patients was found to be significant. Some 82.2% of participants reported experiencing financial difficulties in managing their healthcare costs, with variations between Turkey and Bulgaria.

According to Table 1, which presents financial challenges in accessing mental healthcare, participants expressed concerns regarding the lack of insurance coverage for mental health services, particularly in Bulgaria, where only 27.4% of participants received government support for such care, compared with 39.3% in Turkey. Many respondents reported delaying or avoiding mental health treatment because of high costs, exacerbating both their psychological and ophthalmic conditions.

**Table 1:** Financial challenges in accessing mental healthcare

Financial burden indicator	Turkey (%)	Bulgaria (%)
Difficulty affording mental healthcare	78.5	85.9
Out-of-pocket expenses for mental health services	63.4	72.1
Missed ophthalmic appointments because of financial issues	36.8	53.3
Use of government support for mental healthcare	39.3	27.4

Inferential analyses confirmed significant cross-country differences in financial burden indicators. Participants in Bulgaria were significantly more likely to report difficulty affording mental healthcare compared with Turkish participants (*χ* ^2^(1, *N* = 214) = 4.16, *p* = 0.041). Out-of-pocket expenses were also more frequently reported in Bulgaria (*χ* ^2^ = 3.87, *p* = 0.049), and missed ophthalmic appointments because of financial constraints were significantly more prevalent among Bulgarian patients (*χ* ^2^ = 6.12, *p* = 0.013). These findings are consistent with narrative accounts of greater financial fragility in the Bulgarian healthcare system and reinforce the need for country-specific financial interventions.

To better understand variations in mental health burden, we disaggregated the results by different ophthalmic conditions, including glaucoma, diabetic retinopathy, corneal disorders and retinal diseases. Table 2 presents the prevalence of moderate-to-severe depression (PHQ-9 ≥10) and anxiety (GAD-7 ≥10) across these conditions.

**Table 2:** Mental health symptoms by ophthalmic condition

Ophthalmic condition	Moderate–severe depression (%)	Moderate–severe anxiety (%)
Glaucoma	57.8	52.3
Diabetic retinopathy	64.5	60.8
Corneal Disorders	46.9	44.2
Retinal diseases (e.g. age-related macular degeneration)	62.1	58.4

To assess whether rates of moderate-to-severe depression and anxiety differed significantly across ophthalmic conditions, chi-squared tests were conducted. For depression (PHQ-9 ≥10), the association between condition and prevalence was statistically significant (*χ* ^2^(3, *N* = 214) = 8.43, *p* = 0.038). Similarly, for anxiety (GAD-7 ≥10), there was a significant association with ophthalmic condition (*χ* ^2^(3, *N* = 214) = 7.92, *p* = 0.048). Post hoc pairwise comparisons with Bonferroni correction revealed that patients with diabetic retinopathy and retinal diseases were significantly more likely to report elevated psychological distress than those with corneal disorders. These findings support earlier qualitative insights suggesting that prognosis and reversibility of visual impairment may influence psychological vulnerability.

The findings of this study highlight significant variations in mental health distress among ophthalmic patients, depending on the specific condition they experience. Patients diagnosed with diabetic retinopathy and retinal diseases exhibited the highest levels of mental health distress. This is likely attributed to the progressive and often irreversible nature of these conditions, which can result in severe vision loss or complete blindness over time. The psychological burden associated with these diseases stems from the uncertainty of prognosis, long-term disability and limitations in treatment options, leading to increased levels of depression and anxiety among affected individuals. Similarly, patients with glaucoma reported elevated levels of depression and anxiety, potentially because of the gradual yet permanent nature of vision loss caused by the disease. Unlike sudden vision impairment, glaucoma progresses silently, often without noticeable symptoms in the early stages. This unpredictable decline in vision can lead to feelings of helplessness and fear, as patients struggle to anticipate when and how their sight will deteriorate. The chronic stress of managing intraocular pressure, adhering to lifelong treatments and the risk of eventual blindness further contributes to the mental health burden among glaucoma patients. In contrast, individuals diagnosed with corneal disorders reported lower levels of mental health distress compared with those with retinal or glaucoma-related conditions. This difference is likely because of the better treatment options and higher probabilities of functional recovery available for corneal disorders. Procedures such as corneal transplants, corrective surgeries and advanced therapeutic interventions offer many patients a realistic chance of restoring or significantly improving their vision. As a result, the psychological impact is less severe, as these individuals can maintain hope for visual rehabilitation and experience less anxiety regarding permanent blindness. These findings emphasise the strong correlation between disease prognosis and mental health distress, highlighting the need for targeted psychological support tailored to specific ophthalmic conditions. By recognising these variations, healthcare providers can better address the unique emotional and psychological challenges faced by patients with different types of visual impairments.

The impact of untreated mental health conditions on the quality of life of ophthalmic patients was profound, as revealed through responses to open-ended questions. Three major themes emerged, each highlighting the challenges faced by visually impaired individuals in their daily activities, social interactions and economic participation. Loss of independence, social isolation and employment difficulties were particularly pronounced among patients with progressive eye diseases, such as diabetic retinopathy and glaucoma, which further exacerbated their psychological distress and financial burden.

One of the most significant challenges reported was the loss of independence, particularly among individuals aged 50 and above. Many participants described increasing difficulty in performing essential daily tasks such as reading, cooking, managing household chores and using public transportation. The inability to complete these routine activities without assistance led to a greater sense of helplessness and frustration, which in turn contributed to higher depressive symptoms. This issue was especially severe among diabetic retinopathy and glaucoma patients, whose conditions progressively limit their ability to function independently. As vision deteriorated, many participants became more dependent on caregivers, further reinforcing feelings of loss of control over their lives.

Social isolation also emerged as a major concern, with 72.5% of respondents reporting a significant reduction in social engagement following the decline in their vision. The impact of social withdrawal was even more pronounced in Bulgaria (78.3%) compared with Turkey (66.7%), suggesting potential cultural and healthcare system differences in addressing the psychosocial needs of visually impaired individuals. Many respondents mentioned that their social circles had shrunk, as they no longer felt comfortable participating in gatherings, attending public events or engaging in recreational activities. The fear of falling, difficulty recognising faces and the frustration of navigating unfamiliar spaces contributed to their reluctance to interact socially. Over time, this isolation led to heightened anxiety, loneliness and, in some cases, depressive symptoms, emphasising the need for better social support systems for visually impaired individuals.

A third major theme was the impact of vision impairment on employment and economic productivity. Among respondents who were employed before experiencing significant vision loss, 30.4% stated that they had either reduced their working hours or quit their jobs because of their condition. This trend was especially notable among diabetic retinopathy patients, who reported frequent medical visits, difficulty managing treatment regimens and an inability to perform detail-oriented tasks. Many individuals struggled to maintain stable employment, particularly in professions that required fine motor skills, prolonged screen time or frequent travel. The economic consequences of vision impairment, when coupled with mental health disorders, further intensified financial strain, as individuals faced reduced income, increased medical expenses and fewer job opportunities.

The thematic patterns are further illuminated by representative participant voices (all identifiers removed for anonymity):

- ‘I stopped taking the bus because I can’t read the route numbers anymore. Every trip now depends on my son’s schedule, and it makes me feel useless.’ – Female, 66, diabetic retinopathy, Turkey
- ‘Even if I could afford the psychologist, the waiting list is four months. By then my vision may be worse and so will my mood.’ – Male, 59, retinal disease, Bulgaria
- ‘People think you’re weak if you talk about nerves. I’d rather say my eye hurts than admit I’m anxious.’ – Female, 52, glaucoma, Bulgaria
- ‘Since the corneal transplant was possible, I keep hope. That hope is the main reason I haven’t fallen into depression.’ – Male, 48, corneal disorder, Turkey

These quotations cement how financial strain, health system delays and disease prognosis interact with patients’ emotional experience.

These findings underscore the multi-dimensional impact of vision impairment on quality of life, illustrating how loss of independence, social isolation and employment struggles collectively contribute to the mental health burden of ophthalmic patients. In addition, the cross-country differences in social isolation highlight the importance of cultural attitudes, healthcare accessibility and community support in shaping the well-being of visually impaired individuals. Addressing these issues requires a comprehensive approach that integrates mental health services, occupational support and community-based interventions to improve the overall quality of life for affected individuals.

Barriers to accessing mental healthcare among ophthalmic patients varied significantly based on specific eye conditions and country-specific healthcare challenges. The study revealed several themes that highlight the unique difficulties faced by patients with glaucoma, diabetic retinopathy, corneal disorders and retinal diseases. These barriers ranged from psychological concerns and financial limitations to systemic issues such as long waiting times, all of which contributed to the underutilisation of mental health services among ophthalmic patients.

For glaucoma patients, the primary barrier to seeking mental healthcare was fear of long-term disability and uncertainty about vision loss. Many patients, particularly in Turkey (69.5%), expressed high levels of anxiety about the progressive and often irreversible nature of their condition. This uncertainty created chronic psychological distress, making many patients reluctant to seek mental health support, because of denial, avoidance or a preference to focus solely on ophthalmic treatments. The progressive loss of peripheral vision without immediate symptoms further reinforced feelings of helplessness, as patients struggled with the unpredictability of their future visual capabilities. Previous studies have also highlighted that fear of blindness and loss of autonomy are among the most distressing aspects of glaucoma,^12^ supporting the findings of this study.

Among diabetic retinopathy patients, financial burden was identified as the most significant obstacle to accessing mental health services. This was especially pronounced in Bulgaria, where 83.2% of patients reported financial constraints in seeking psychiatric care, compared with 65.4% in Turkey. Since diabetic retinopathy requires frequent ophthalmic treatments, medical visits and costly interventions, many patients reported prioritising their vision-related expenses over mental healthcare. The financial strain associated with managing diabetes itself, in addition to vision-related complications, left little room for mental health treatment. The out-of-pocket costs for psychiatric consultations and medications in Bulgaria were particularly challenging for those without comprehensive insurance, reinforcing findings from previous research that economic constraints remain a major barrier to mental healthcare access in middle-income countries.^13^

Patients with corneal disorders faced a different challenge: lack of awareness about mental health symptoms. Many individuals with corneal conditions did not recognise depression or anxiety as serious concerns, often considering them secondary to their vision-related issues. This perception may stem from the fact that corneal disorders often have more promising treatment options, such as corneal transplants or corrective surgeries, which offer a higher likelihood of functional recovery. As a result, these patients were less likely to perceive a need for mental health intervention, even when experiencing psychological distress related to vision impairment. This lack of awareness suggests that more proactive screening and patient education about mental health in ophthalmic settings could help address undiagnosed psychiatric conditions in this population.

For retinal disease patients, the most significant barrier was long waiting times for psychiatric care, particularly in Bulgaria. Patients with retinal diseases in Bulgaria reported an average wait time of 4.3 months to receive psychiatric care, compared with 1.8 months in Turkey. The long wait times discouraged many patients from seeking help at all, as they felt overwhelmed by the bureaucracy and lengthy referral processes within the healthcare system. Since retinal diseases often involve sudden and severe visual deterioration, patients required immediate psychological support to cope with vision loss and its associated emotional impact. However, the lack of timely access to mental healthcare exacerbated feelings of distress, helplessness and frustration. These findings align with broader healthcare studies that indicate prolonged wait times for mental health services can lead to worsening psychiatric symptoms and higher drop-out rates from treatment.^14^

These findings underscore the complex barriers to mental healthcare access faced by ophthalmic patients. The variation across eye conditions and country contexts highlights the need for targeted interventions that address both systemic and condition-specific challenges. As shown in Table 3, different ophthalmic conditions were associated with distinct barriers to mental healthcare access, with financial constraints being most prevalent among diabetic retinopathy patients (72.3%), while long waiting times posed the greatest challenge for retinal disease patients (55.7%). The table provides a comparative breakdown of these barriers by condition, illustrating the urgent need for condition-specific mental health interventions.

**Table 3:** Barriers to seeking mental healthcare by ophthalmic condition

Barrier by condition	Glaucoma (%)	Diabetic retinopathy (%)	Corneal disorders (%)	Retinal diseases (%)
Fear of stigma	45.1	40.6	33.2	49.8
Financial constraints	59.4	72.3	48.7	66.1
No mental health referral in ophthalmic clinics	70.8	74.5	61.9	76.4
Long waiting times	36.5	41.2	32.8	55.7

Chi-squared analyses further explored whether barriers to mental healthcare differed by ophthalmic diagnosis. Long waiting times were significantly more likely to be reported by patients with retinal diseases compared with other groups (*χ* ^2^(3, *N* = 214) = 9.34, *p* = 0.025). Financial constraints also varied by condition and approached statistical significance (*χ* ^2^ = 7.56, *p* = 0.056), with the highest levels reported among diabetic retinopathy and retinal disease patients. Fear of stigma did not differ significantly across conditions (*χ* ^2^ = 5.81, *p* = 0.12), although it remained a salient theme in qualitative responses. These patterns align with the condition-specific stressors described in earlier sections and help quantify differential access barriers faced by ophthalmic sub-populations.

The study revealed notable differences between Turkey and Bulgaria regarding access to mental health services for ophthalmic patients. Bulgarian patients consistently experienced greater financial strain in obtaining mental healthcare across all ophthalmic conditions. This was largely because of higher out-of-pocket expenses, lower insurance coverage and limited government support for mental health services. In contrast, Turkey’s more extensive healthcare system provided broader coverage, alleviating some of the financial pressure. However, even in Turkey, patients with retinal diseases and diabetic retinopathy still faced substantial out-of-pocket expenses, which discouraged many from seeking psychiatric care. These findings align with previous research indicating that financial barriers remain one of the most significant obstacles to mental healthcare access in middle-income countries.^13^

Another key difference between the two countries was the level of mental health stigma, which was more pronounced in Bulgaria. Patients with glaucoma and retinal diseases in Bulgaria were particularly hesitant to seek psychological help, often perceiving mental health issues as a secondary concern or a personal weakness. This stigma contributed to lower mental health service utilisation rates and a greater reliance on informal coping mechanisms, such as avoiding discussions about their emotional distress. Conversely, while mental health stigma was still present in Turkey, awareness campaigns and mental health advocacy efforts have led to slightly better recognition and acceptance of psychiatric care in recent years. Previous studies on mental health perceptions in Eastern Europe have also found that societal attitudes towards mental health remain a significant barrier to treatment-seeking behaviours in post-socialist nations.^15^

The sharper financial and stigma-related barriers observed in Bulgaria likely reflect intersecting historical and structural factors. Bulgaria’s post-socialist health insurance model remains fragmented, with a higher proportion of out-of-pocket payments for out-patient psychiatry and fewer practising psychiatrists per capita than Turkey.^16^ In contrast, Turkey’s Health Transformation Programme (2003–2020) expanded social security coverage, partially normalising help-seeking.^17^ Culturally, qualitative work suggests that in Bulgaria mental illness is still closely associated with the legacy of institutional asylums, fuelling fear of labelling, whereas recent Turkish media campaigns have framed depression and anxiety as treatable, everyday conditions. These systemic and cultural divergences offer a plausible explanation for the statistically and narratively documented gaps in affordability and stigma, underscoring the need for country-tailored policy solutions rather than a one-size-fits-all regional approach.

One of the most significant disparities between the two countries was waiting times for psychiatric services. In Bulgaria, retinal disease patients faced the longest wait times, with an average delay of 4.3 months before receiving mental healthcare, compared with 1.8 months in Turkey. These long wait times led to delayed diagnoses, worsening psychiatric symptoms and increased frustration among patients. The bureaucratic inefficiencies within the Bulgarian healthcare system contributed to lengthy referral processes and limited availability of mental health professionals, particularly in rural areas. In contrast, Turkey had a slightly more efficient mental healthcare referral system, although wait times still posed a challenge, especially for specialised psychiatric services. Long wait times for mental healthcare services have been widely documented as a critical issue worldwide, with studies emphasising that delays in mental health treatment often exacerbate symptoms and decrease the likelihood of successful intervention.^14^

The study highlights several critical insights into the relationship among ophthalmic conditions, mental health burdens and economic barriers. One of the most striking findings was that depression and anxiety levels were highest among diabetic retinopathy and retinal disease patients. This was likely because of the progressive and irreversible nature of these conditions, which limit treatment options and create uncertainty about long-term vision outcomes. The psychological distress associated with these conditions was further compounded by financial constraints and social isolation, leading to heightened rates of anxiety and depression among affected individuals.

Financial constraints disproportionately affected diabetic retinopathy patients, particularly in Bulgaria, where 83.2% of patients cited financial difficulties as a major barrier to seeking psychiatric care. Since diabetic retinopathy requires continuous medical intervention, including frequent check-ups, laser treatments and medications, many patients prioritised their ophthalmic care over mental health support because of limited financial resources. This financial burden significantly reduced mental healthcare utilisation rates, reinforcing the need for policy interventions aimed at expanding insurance coverage and providing financial assistance for mental health services in middle-income countries.

Another key finding was the stronger presence of mental health stigma in Bulgaria, particularly among glaucoma and retinal disease patients. Many Bulgarian participants were reluctant to seek psychological help, often dismissing symptoms of depression and anxiety as inevitable consequences of vision loss rather than treatable conditions. This attitude contributed to low engagement with psychiatric services and reduced adherence to mental health treatment plans. In contrast, Turkish patients were slightly more open to seeking mental healthcare, although stigma remained a significant obstacle to treatment-seeking behaviours. Addressing mental health stigma through public education campaigns, community-based mental health initiatives and increased collaboration between ophthalmologists and mental health professionals could improve mental health outcomes for visually impaired individuals in both countries.

Finally, retinal disease patients in Bulgaria faced the longest waiting times for mental healthcare services, further exacerbating their psychological distress and overall healthcare experience. The delay in accessing psychiatric care left many patients without adequate support during critical periods of vision deterioration, increasing their risk of severe depression and anxiety. The findings emphasise the need for healthcare policy reforms that improve access to timely psychiatric care, particularly for high-risk patient groups, such as those with progressive ophthalmic conditions. Addressing waiting times, financial barriers and mental health stigma through integrated healthcare models and policy-driven interventions could significantly enhance the quality of life and mental well-being of ophthalmic patients in Turkey, Bulgaria and beyond.

## Discussion

The findings of this study provide a comprehensive understanding of the economic toll of untreated mental health disorders in ophthalmic patients across two different healthcare systems, Turkey and Bulgaria. The high prevalence of moderate-to-severe depression and anxiety among participants highlights the significant mental health burden faced by individuals with vision impairment. Our results align with previous studies that suggest that individuals with chronic ophthalmic conditions, particularly those with progressive and irreversible vision loss, are at heightened risk for developing psychiatric comorbidities.^3,4^ The financial burden of mental healthcare was a significant factor influencing access to treatment, with a larger proportion of Bulgarian patients reporting difficulty affording care compared with their Turkish counterparts.

One of the most significant takeaways from this study is the financial burden faced by ophthalmic patients dealing with untreated mental health disorders. The economic costs of depression and anxiety have been well documented in various studies,^2^ but when compounded with vision impairment, the impact appears even more severe. Our results indicate that 82.2% of participants experienced financial difficulties related to their healthcare, with Bulgarian respondents struggling more than their Turkish counterparts. This finding aligns with research showing that financial constraints are one of the primary barriers to accessing mental health services worldwide.^14^

A higher reliance on out-of-pocket payments in Bulgaria explains some of these discrepancies. In contrast, Turkey, with a relatively more extensive insurance system, has higher coverage for mental healthcare, but gaps still remain, particularly among ophthalmic patients who might not see mental healthcare as a priority until symptoms become severe. Other studies highlight similar trends, noting that individuals in middle-income countries often struggle with out-of-pocket costs for mental healthcare, despite public healthcare provisions.^13^

Our study found notable differences in the mental health burden across various ophthalmic conditions. Patients with diabetic retinopathy and retinal diseases reported the highest levels of depression and anxiety, with 64.5% of diabetic retinopathy patients showing moderate-to-severe depressive symptoms (PHQ-9 ≥10). These findings are in line with previous research indicating that diabetic retinopathy patients experience significant psychological distress because of the progressive and potentially debilitating nature of the disease.^18^ Similarly, individuals with glaucoma exhibited high anxiety levels (52.3%), which is consistent with studies highlighting that the gradual and often asymptomatic progression of glaucoma contributes to significant psychological stress.^12^ In addition, it was also reported that depression was more common in individuals with moderate-to-severe diabetic retinopathy (16%) than in those without the condition (7%).^19^

Conversely, individuals with corneal disorders demonstrated comparatively lower rates of depression and anxiety. This could be attributed to greater treatment options and higher recovery probabilities for corneal disorders, as opposed to the often irreversible nature of retinal diseases. Some research supports this notion, suggesting that perceived reversibility of eye conditions plays a role in determining psychological impact.^20^

The lack of integration between ophthalmic and mental health services is a significant concern in healthcare systems. Many participants in our study reported that their ophthalmologists did not enquire about mental health concerns or provide referrals to psychiatric care. This reflects a broader issue where mental and physical health are often treated separately, despite clear evidence of their interconnection. Previous research has emphasised that mental disorders increase the risk for communicable and non-communicable diseases and contribute to unintentional and intentional injury, underscoring the necessity for integrated care.^21^ Furthermore, some have also highlighted the importance of integrating mental health screening in specialty clinics, including ophthalmology, to address the needs of high-risk patients.^22^ These researchers developed a framework for automating psychiatric distress screening in ophthalmology clinics using an electronic health record-derived artificial intelligence algorithm, aiming to facilitate early identification and intervention. These studies collectively advocate for a more holistic approach to healthcare, where mental health is considered an integral component of patient care across all medical specialties.

Stigma associated with seeking mental health support emerged as a critical issue in our study, particularly in Bulgaria, where 48.5% of respondents identified it as a major barrier to care. This finding aligns with existing literature indicating that cultural attitudes towards mental illness significantly influence healthcare utilisation. In contrast, while stigma was still present in Turkey, it was reported at slightly lower rates. This difference may reflect the impact of public awareness campaigns and increased mental health literacy in recent years. For instance, a study analysing Turkish Twitter content found that terms such as ’schizophrenia’ and ‘psychosis’ were frequently misused and associated with negative attitudes, suggesting ongoing stigma in Turkish society. However, the same study noted instances of medically accurate information and supportive statements, indicating a growing awareness and understanding of mental health issues. These findings suggest that while stigma remains a barrier in Turkey, efforts to improve public perception and knowledge about mental health may be contributing to a gradual reduction in stigmatising attitudes.^23^

Based on these findings, several policy recommendations emerge.

- Routine mental health screening in ophthalmology clinics:
  - ophthalmologists and optometrists should implement routine depression and anxiety screenings using standardised tools such as the PHQ-9 and GAD-7;
  - integrated care pathways should be established to ensure patients with positive screenings receive timely referrals to mental health professionals.
- Financial support for mental health services:
  - in Bulgaria, government subsidies or insurance coverage should be expanded to reduce out-of-pocket costs for mental healthcare;
  - in Turkey, enhancing insurance schemes to cover mental health services within ophthalmic settings can improve accessibility.
- Educational campaigns to reduce stigma:
  - public awareness programmes targeting stigma reduction in mental healthcare should be expanded, particularly in Bulgaria;
  - providing culturally appropriate mental health education through patient support groups and healthcare provider training can improve treatment adherence.
- Research on long-term economic impacts:
  - future research should examine the long-term economic consequences of untreated mental health disorders in ophthalmic patients, focusing on productivity losses, healthcare utilisation and cost-effectiveness of integrated care models;
  - longitudinal studies comparing different healthcare models can provide deeper insights into effective interventions.

Despite the strengths of this study, including a diverse sample across two different healthcare systems and the use of both quantitative and qualitative methodologies, some limitations must be acknowledged. First, the study relied on self-reported measures, which may introduce recall bias or underreporting of symptoms. Second, the sample was limited to two countries, meaning findings may not be generalisable to all middle-income settings. Future studies should expand to other regions to assess cross-cultural variations in the economic burden of mental health disorders among ophthalmic patients.

## Data Availability

Anonymised qualitative data are available upon request from the corresponding author.
